# Main lesions in the central nervous system of dogs due to *Leishmania infantum* infection

**DOI:** 10.1186/s12917-017-1174-5

**Published:** 2017-08-18

**Authors:** Weline Lopes Macau, Joicy Cortez de Sá, Ana Patrícia de Carvalho da Silva, Alessandra Lima Rocha, Renata Mondêgo-Oliveira, Fábio Henrique Evangelista de Andrade, Caroline Magalhães Cunha, Kátia da Silva Calabrese, Ana Lucia Abreu-Silva

**Affiliations:** 1 Center for Biological and Health Sciences, Universidade Federal do Maranhão, São Luis, MA CEP 65080-805 Brazil; 2 Medicine Coordination, Universidade CEUMA, São Luís, MA CEP 65055-000 Brazil; 3 Veterinary School, Universidade Federal do Tocantins, Araguaína, TO CEP 77804-970 Brazil; 4 Department of Pathology, Universidade Estadual do Maranhão, São Luís, MA CEP 65055-000 Brazil; 5 Laboratory of Immunomodulation and Protozoology, Instituto Oswaldo Cruz, Rio de Janeiro, Brazil

**Keywords:** Dog, Encephalon, Histopathology, Immunohistochemistry, *Leishmania infantum*, Polymerase chain reaction

## Abstract

**Background:**

Canine visceral leishmaniasis (CVL) is endemic in São Luís Maranhão/Brazil and it leads a varied clinical picture, including neurological signs.

**Results:**

Histopathological evaluation showed that 14 dogs exhibited pathological alterations in at least one of the analyzed areas. Of these, mononuclear inflammatory reaction was the most frequent, although other lesions, such as hemorrhage, chromatolysis and gliosis were also observed. The presence of *L. infantum* amastigotes was confirmed in eight dogs, identified in four regions: telencephalon, hippocampus, thalamus and caudal colliculus, but only one presented neurological signs. Polymerase chain reaction results detected the DNA of the parasite in 11 samples from seven dogs. The positive areas were the telencephalon, thalamus, hippocampus, cerebellum, caudal and rostral colliculus.

**Conclusion:**

These results reveal that during canine visceral leishmaniasis, the central nervous system may display some alterations, without necessarily exhibiting clinical neurological manifestations. In addition, the *L. infantum* parasite has the ability to cross the blood brain barrier and penetrate the central nervous system.

## Background

Canine visceral leishmaniasis (CVL) is a chronic and progressive disease, characterized by the dissemination of the parasite through various organs. The amastigote forms of *Leishmania,* however, are found mostly in the mononuclear phagocytic system organs, such as the spleen, lymph nodes, liver and bone marrow [1–4].

The blood–brain barrier (BBB) is a dynamic and functional structure that separates the central nervous system from the systemic circulation and is required for maintaining neuronal function [5]. It has been believed for some time that *Leishmania* parasites were unable to cross the BBB. However, in 1996, the presence of *Leishmania donovani* amastigote forms in the cerebrospinal fluid (CSF) was described for first the time in a human patient [6]. Since then, the central nervous system has become the subject of many studies seeking to confirm that *Leishmania* species cross the BBB and cause pathological alterations and clinical neurological signs [8, 10, 11, 20, 22].

A study with dogs positive for visceral leishmaniasis, demonstrated the presence of anti-*Leishmania* antibodies in the CSF, that alterations in the nervous tissue of these animals can occur and this may result in a form of the disease known as “cerebral leishmaniasis” [7]. Experimental studies in mice infected with *Leishmania amazonensis*, associated with the cutaneous form of leishmaniasis in humans, described the occurrence of brain lesions with discrete hyperemia and inflammatory infiltrates in the meninges, composed of mononuclear cells and neutrophils [8].

Another study using an experimental model of infection in hamsters with *Leishmania* sp. identified the dissemination of the parasite in a number of organs. The main finding of the study was the high percentage of animals with parasites in the central nervous system [9].

Neurological signs following natural infection in dogs were described in a case of acute paraplegia associated with vasculitis [10]. In this study, post-mortem examination revealed a large area of hemorrhaging on the nervous tissue of the spinal cord, together with thrombus and a mixed inflammatory cell infiltrate. Many other neurological signs, suggesting the widespread involvement of the central nervous system, had already been observed through a rigorous clinical evaluation. These included convulsions, blindness, signs of vestibular and cerebellar involvement (tilted head, nystagmus, motor incoordination, and tremors), paraparesis, tetraparesis, tetraplegia and myoclonia [11].

Therefore, the aim of this study was to evaluate histopathological alterations and verify the presence of *L. infantum* in specific areas of the encephalon of naturally infected dogs, in order to verify if this parasite results in tropism in a certain region of the central nervous system.

## Methods

### Animals

This study was performed at the Veterinary Hospital of the Universidade Estadual do Maranhão, Brazil, in accordance with the Ethics Committee on Animal Experimentation (protocol number 05/2006). The dogs owners signed and informed consent form. Fifteen male and female mongrel dogs from São Luís Island, an *L. infantum* endemic area took a part of this experiment. All animals were positive for *L. infantum* for direct parasitological examination (bone marrow aspiration) and serological test (ELISA). After clinical evaluation, the animals received 2% xylazine as premedication (0.1 mL/kg intravenously) and 2.5% thiopental as an anesthetic. After a latency period of 5 min, euthanasia was performed by injecting 10% potassium chloride intravenously. Necropsy was then carried out for brain removal.

### Histopathological and immunohistochemistry analysis

The entire encephala of the dogs were removed and fixed in 10% buffered formalin for 24 h. After that, fragments of the cerebellum, cerebellar peduncle, rostral colliculus, caudal colliculus, thalamus, hippocampus, and telencephalon were obtained, submitted to histological processing, and stained by the standard Hematoxilyn-eosin method. For immunohistochemical analysis, sections of 5 μm were fixed on silanized slides and the streptavidin-peroxidase method was performed [12].

### DNA extraction from paraffin-embedded samples

Sections (10 μm) of each tissue sample were collected in microtubes to remove paraffin with xylol. Next, the tissues were rehydrated using decreasing concentrations of ethanol and the supernatant was removed. A total of 200 μL of lysis buffer and 20 μL of proteinase K (20 mg/ml) were added as described by Pikor et al. [13]. The extraction was carried out using the phenol-chloroform-isoamylic alcohol method. The extracted DNA samples were re-suspended in 15 μL of ultrapure water and stored at −20 °C until use as previously described by de Lima [14].

### Polymerase chain reaction (PCR)

The extracted DNA was amplified in a thermocycler using the primers RV1 (sense; 5′-CTTTTCTGGTCCCGCGGGTAG-3′) and RV2 (antisense; 5’CCACCTGGCCTATTTTACACCA-3′). This targets a conserved region of *L. (L.) infantum* kDNA and produces a fragment of approximately 145 base pairs. Positive and negative controls were used in each reaction. The PCR products were analyzed on 1% agarose gel with a molecular weight marker of 100 bp (1Kb DNA ladder Promega®) [15].

### Isolation and characterization of the parasites

Parasites were isolated from bone marrow and cultivated in Novy Macneal Nicolle medium (NNN) and posteriorly sent to Laboratory of Immunomodulation and Protozoology/IOC – FIOCRUZ, incubated at 26 to 28 °C and examined weekly. Positive cultures were sent to the National Reference Laboratory for Leishmania (CLIOC) of the Oswaldo Cruz Institute to characterize the *Leishmania* species using the enzyme electrophoresis technique [16].

## Results

The isolated parasites were characterized by the technique of isoenzyme electrophoresis as identified *L. infantum.* The animals used in the present study displayed at least one clinical sign compatible with visceral leishmaniasis, only one animal showed signs of neurological disease. The most frequently observed clinical signs were cutaneous lesions (86.6%) and cachexia (66.6%). In post-mortem evaluation, hepatosplenomegaly and lymphadenopathy, present in 86.6% of the dogs, were the most frequent alterations.

**Fig. 1:**
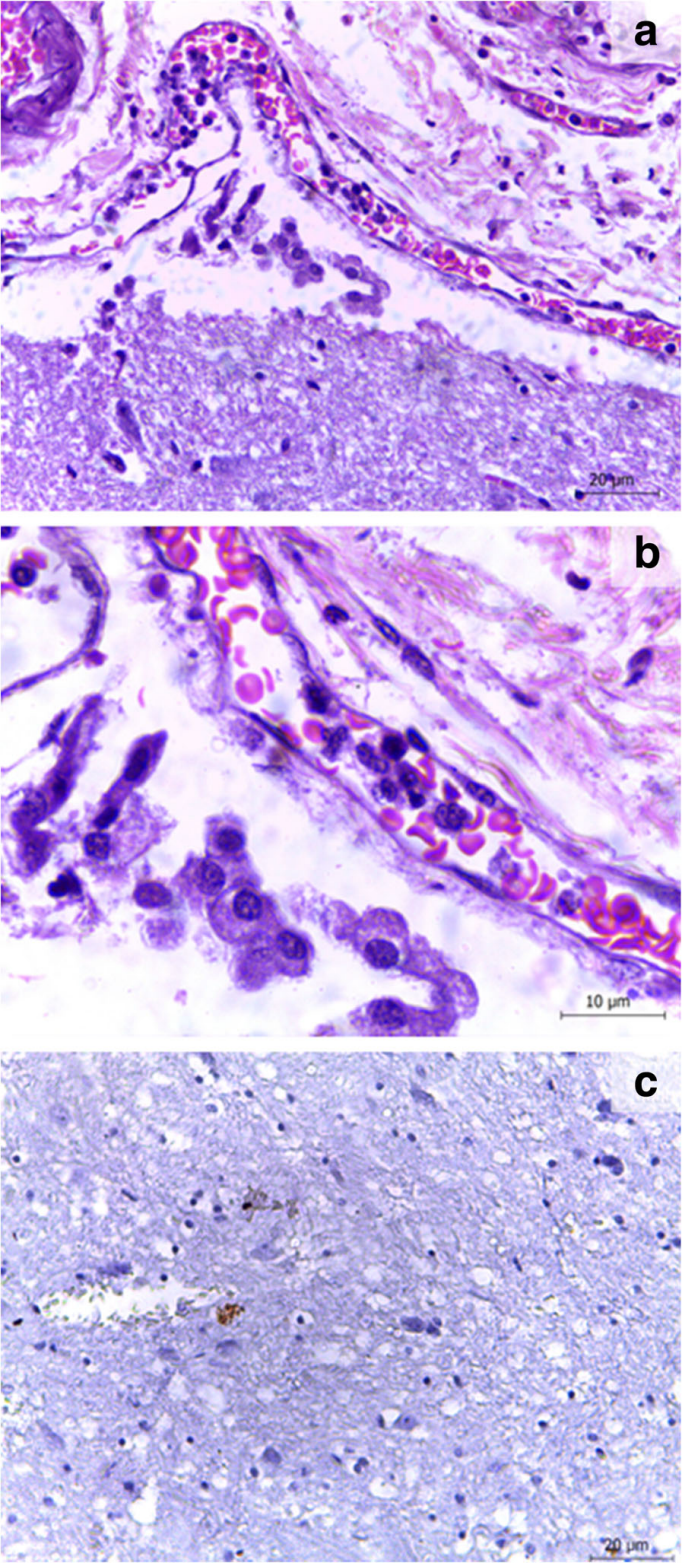
**a** Microscopic lesions in cerebellum. Encephalon of dog with visceral leishmaniasis presenting inflammatory infiltrate with foamy macrophages or Gitter cell in the cerebellum region (bar 20 μm): **b** higher magnification (bar 10 μm). Hematoxylin and eosin stain. **c** Encephalon of dog with visceral leishmaniasis. Rostral colliculus region marked amastigote forms (arrow) Immunohistochemistry (bar 20 μm)

Histopathological analysis revealed that 93.3% of the animals had lesions in at least two of the analyzed brain regions. These lesions were distributed in the telencephalon, hippocampus, rostral colliculus and cerebellum. Lymphoplasmocitary inflammatory reaction was the most frequent lesion (37.1%) associated with rare amastigotes, Gitter cells also were frequent among inflammatory cells (Fig. 1. hemorrhages (10.4%), gliosis (4.7%) and chromatolysis (3.8%) were also observed.

Immunohistochemical analysis detected amastigote forms in seven dogs. The amastigotes were associated with an inflammatory reaction in all the animals (Fig. 1c). Parasites were found in four different regions: the thalamus (*n* = 3), telencephalon (*n* = 3), hippocampus (*n* = 2) and caudal colliculus (*n* = 2).

Polymerase chain reaction detected parasite DNA in 11 samples from seven dogs. The positive encephalic regions were the telencephalon (*n* = 3); thalamus (*n* = 2), hippocampus (*n* = 2); cerebellum (*n* = 2); caudal colliculus (*n* = 1) and rostral colliculus (*n* = 1). All positive samples in PCR also presented some histopathological changes, such as inflammatory reaction composed by mononuclear cells.

## Discussion

During *Leishmania* infection the BBB may be damaged, allowing that amastigotes reach brain parenchyma, where an intensive inflammatory reaction can be observed. The mechanisms by which this process occurs remain unknown, however, some intracellular bacteria, such as *Listeria monocytogenes* seem to cross the BBB inside leukocytes, a mechanism known as “Trojan horse” [17, 18]. Abreu-Silva et al. [8] suggest that *Leishmania* uses this strategy to penetrate the brain parenchyma.

Inflammatory reaction during canine visceral leishmaniasis was also observed by Ikeda et al. [11], who analyzed brains of dogs with and without neurological signs and described degenerative alterations with neuronophagia, gliosis, leptominingitis, vascular congestion and the presence of a perivascular lymphoplasmacytic infiltrate and areas of micro-hemorrhages.

Melo et al. [19] evaluated the presence of leukocytes in the brains of dogs with VL, and observed significant inflammatory lesions consisting of lymphocytes, macrophages, plasma cells and some neutrophils. An immunohistochemical study showed that CD3+ T lymphocytes are important components of the inflammatory infiltrate present in the choroid plexus, meninges and perivascular brain parenchyma. Moreover, the cytokine profile identified high expressions of IL-1β and TNF-α, which are considered key factors for the beginning, maintenance and persistence of inflammation [20].

*Leishmania* amastigotes in the CNS of dogs with visceral leishmaniasis were previously described by [21, 22]. Other studies, despite the observation of histopathological alterations in the CNS of dogs during visceral leishmaniasis, failed to verify the presence of *Leishmania* amastigotes [11, 19, 20, 23–25]. Some authors explain these contradictory findings by the chronicity of the disease, arguing that the presence of *Leishmania* in the CNS has generally been demonstrated in dogs with an earlier infection, either because of delayed diagnosis or by the choice of treatment for the infected dogs, as in two studies in the Mediterranean region [11, 24]. In fact, here we observed that animals that presented amastigote forms in the CNS had clinical signs such as cachexia and the presence of skin lesions, observed in the chronic phase.

## Conclusion

Dogs naturally infected *L.* infantum can present alterations in the CNS due mononuclear inflammatory infiltrate. Furthermore, *Leishmania* parasites have unknown mechanisms that allow their entrance into the CNS, and show no preference for a specific region of the system.

## Abbreviations

BBB: Blood–brain barrier
CNS: Central nervous system
CSF: Cerebrospinal fluid
CVL: Canine visceral leishmaniasis
PCR: Polymerase chain reaction

## References

[1] Alvar J, Cañavate C, Molina R, Moreno J, Nieto J (2004). Canine leishmaniasis. Adv Parasitol.

[2] Baneth G, Koutinas AF, Solano-Gallego L, Bourdeau P, Ferrer L (2008). Canine leishmaniasis – new concepts and insights on an expanding zoonosis: part one. Trends Parasitol.

[3] Giunchetti RC, Martins-Filho OA, Carneiro CM, Mayrink W, Marques MJ, Tafuri WL (2008). Histopathology, parasite density and cell phenotypes of the popliteal lymphnode in canine visceral leishmaniasis. Vet Immunol Immunopathol.

[4] Giunchetti RC, Mayrink W, Carneiro CM, Corrêa-Oliveira R, Martins-Filho OA, Marques MJ (2008). Histopathological and immunohistochemical investigations of the hepatic compartment associated with parasitism and serum biochemical changes in canine visceral leishmaniasis. Res Vet Sci.

[5] Persidsky Y, Ramirez SH, Haorah J, Kanmogne GD (2006). Blood–brain barrier: structural components and function under physiologic and pathologic conditions. J NeuroImmune Pharmacol.

[6] Prasad LS, Sen S (1996). Migration of *Leishmania donovani* amastigotes in the cerebrospinal fluid. Am. J. Trop. Med. Hyg..

[7] Garcia-Alonso M, Nieto CG, Blanco A, Requena JM, Alonso C, Navarrete I (1996). Presence of antibodies in the aqueous humour and cerebrospinal fluid during *Leishmania* infections in dogs. Pathological features at the central nervous system. Parasite Immunol.

[8] Abreu-Silva AL, Calabrese KS, Tedesco RC, Mortara RA, Costa SCG (2003). Central nervous system involvement in experimental infection with *Leishmania* (*Leishmania*) *amazonensis*. Am J Trop Med Hyg.

[9] Oliveira E, Oshiro ET, Pinto R, Castro BC, Daniel KB, Oliveira JM (2011). Presence of amastigotes in the central nervous system of hamsters infected with *Leishmania* sp. Rev Bras Parasitol Vet.

[10] Font A, Mascort J, Altimira J, Closa JM, Vilafranca M (2004). Acute paraplegia associated with vasculitis in a dog with leishmaniasis. J Small Anim Pract.

[11] Ikeda FA, Laurenti MD, Corbett CE, Feitosa MM, Machado GF, Perry SHV (2007). Histological and immunohistochemical study of the central nervous system of dogs naturally infected by *Leishmania* (*Leishmania*) *infantum*. Braz J Vet Res Anim Sci.

[12] Tafuri WL, Santos RL, Arantes RME, Gonçalves R, Melo MN, Michalik MSM, Tafuri WL (2004). An alternative immunohistochemical method for detecting *Leishmania* amastigotes in paraffin-embedded canine tissues. J Immunol Methods.

[13] Pikor LA, Enfield KSS, Cameron H, Lam WL (2011). DNA extraction from paraffin embedded material for genetic and epigenetic. Jo VE.

[14] de Lima AC, Zampieri RA, Tomokane TY, Laurenti MD, Silveira FT, Corbett CE, Floeter-Winter LM, Gomes CM (2011). *Leishmania s*p. identification by PCR associated with sequencing of target SSU rDNA in paraffin-embedded skin samples stored for more than 30 years. Parasitol Res.

[15] Lachaud L, Marchergui-Hammami S, Chabbert E, Dereure J, Dedet JP, Bastien P (2002). Comparison of six PCR methods using peripheral blood for detection of canine visceral Leishmaniasis. J Clin Microbiol.

[16] Cupolillo E, Grimaldi G, Momen H (1994). A general classification of new world *Leishmania* using numerical zymotaxonomy. Am J Trop Med Hyg.

[17] Drevets DA (1999). Dissemination of *Listeria monocytogenes* by infected phagocytes *Infect*. Immun.

[18] Masocha W, Kristensson K (2012). Passage of parasites across the blood–brain barrier. Virulence.

[19] Melo GD, Machado GF (2009). Choroid plexus involvement in dogs with spontaneous visceral leishmaniasis: a histopathological investigation. Braz J Vet Pathol.

[20] Melo GD, Seraguci TF, Schweigert A, Silva JES, Grano FG, Peiró JR (2013). Pro-inflammatory cytokines predominate in the brains of dogs with visceral leishmaniasis: a natural model of neuroinflammation during systemic parasitic infection. Vet Parasitol.

[21] Viñuelas J, García-Alonso M, Ferrando L, Navarrete I, Molano I, Mirón C (2001). Meningeal leishmaniasis induced by *Leishmania infantum* in naturally infected dogs. Vet Parasitol.

[22] Marquez M, Pedregosa JR, López J, Marco-Salazar P, Fondevila D, Pumarola M. Leishmania amastigotes in the central nervous system of a naturally infected dog. J Vet Diagn Investig. 2012;25(1):142-6. 10.1177/1040638712466728 23166183

[23] Lima VMF, Gonçalves ME, Ikeda FA, Luvizotto MCR, Feitosa MM (2003). Anti-*Leishmania* antibodies in cerebrospinal fuid from dogs with visceral leishmaniasis. Bras J Med Bio Res.

[24] Melo GD, Marcondes M, Vasconcelos RO, Machado GF (2009). Leukocyte entry into the CNS of *Leishmania chagasi* naturally infected dogs. Vet Parasitol.

[25] Melo GD, Machado GF (2011). Glial reactivity in dogs with visceral leishmaniasis: correlation with T lymphocyte infiltration and with cerebrospinal fluid anti-*Leishmania* antibody titres. Cell Tissue Res.

